# Effects of flowering period and cultivation practices on volatile organic compounds in Sanqi flowers

**DOI:** 10.3389/fpls.2026.1761183

**Published:** 2026-02-06

**Authors:** Fan Yang, Yue Li, Tongning Yi, Jingying Hei, Biao Wang, Xiahong He, Shu Wang

**Affiliations:** 1 College of Landscape Architecture and Horticulture, Southwest Forestry University, Kunming, China; 2 Department of Endocrinology, the Affiliated Hospital of Liaoning University of Traditional Chinese Medicine, Shenyang, China; 3 Department of Biochemistry and Molecular Biology, School of Life Sciences, China Medical University, Shenyang, China; 4 Yunnan Provincial Key Laboratory for Conservation and Utilization of In-forest Resource, Southwest Forestry University, Kunming, China

**Keywords:** food additives, gas chromatography–mass spectrometry, headspace solid-phase microextraction, RDA analysis, Sanqi flowers, sesquiterpenes, VOCs

## Abstract

Sanqi flower holds broad application prospects in the food industry due to its rich bioactive components and potential health benefits. However, limited information is available regarding the variation of volatile organic compounds (VOCs) in the Sanqi flowers within Sanqi–*Pinus armandii* (SPA) and Sanqi–*Pinus yunnanensis* (SPY) agroforestry systems. Here, the VOCs in Sanqi flowers were compared and analyzed using headspace–solid phase microextraction (HS-SPME) and gas chromatography–mass spectrometry (GC-MS). A total of 60 and 55 VOCs were identified in the Sanqi flowers obtained from the SPY and SPA agroforestry systems, respectively. Terpenes were found to be the predominant components. Moreover, Germacrene D (ranging from 21.35–26.24% to 15.46–24.41%), β-Ocimene (ranging from 21.28–6.60% to 1.21–22.48%) and β-Elemene (ranging from 11.72–13.52% to 9.15–18.77%) were the most abundant in the SPY and SPA systems. Hierarchical clustering analysis (HCA) showed that the VOCs in the Sanqi flowers within the SPY system were clustered into one group. Principal component analysis (PCA) indicated that the VOCs in Sanqi flowers were primarily influenced by the cultivation system. Furthermore, trans-Nerolidol, (E)-4,8-Dimethylnona-1,3,7-triene and γ-Muurolene from the PYS system and Cubenene, Espatulenol, and Bicyclogermacrene from the PAS system can serve as the distinctive VOCs in Sanqi flowers. Redundancy analysis (RDA) revealed that the main factors affecting the VOCs in Sanqi flowers were humidity, followed by temperature. This study provides a theoretical basis for understanding the impact of different agroforestry systems on the VOCs of Sanqi flowers and offers insights into optimizing cultivation practices to enhance the medicinal edible qualities of Sanqi.

## 1 Introduction

The Sanqi (*Panax Notoginseng* (Burk.) F.H.Chen) flower is often used as a medicinal material, tea drink and food additive, thanks to its unique non-volatile and volatile organic compounds (VOCs) (Zhang et al., 2018; Yang et al., 2017; Chen S. Y. et al., 2022). It demonstrates significant efficacy in regulating the cardiovascular system, exhibiting anti-inflammatory effects, possessing antioxidant properties, providing neuroprotection, and improving metabolic diseases (Huang et al., 2023; Zhou et al., 2019; Liu J. C. et al., 2023). Sanqi flowers have a long history of consumption and are considered an excellent source of vitamins, minerals, proteins and amino acids, making them a promising new food ingredient (Yang et al., 2017). Currently, Sanqi flowers are used to brew tea and to enhance the flavour and health benefits of various liquids, including soups and beverages. They can also be used to flavour dishes such as egg and flower cakes. Ornamental plant floral fragrances repel herbivores, resist pathogens, protect flowers, attract pollinators, facilitate plant communication, and enhance aesthetic appeal (Unsicker et al., 2009; Li et al., 2022) (Unsicker et al., 2009; Li et al., 2022). Medicinal plant floral fragrance not only maintains the aforementioned roles but also releases aromatic compounds signaling flower quality, further elevating therapeutic value (Liu et al., 2024). Consequently, the study of floral fragrance compounds in Sanqi flowers is of great significance, as it can provide insights into their potential health benefits and applications in the food and pharmaceutical industries.

Organic cultivation improves the nutritional value, sensory quality and safety of agricultural products (Maggio et al., 2013), and for medicinal herbs, it boosts key chemical content, reduces pesticide residues, and stabilizes aroma and medicinal effects (Zhang et al., 2025). For instance, organic management elevates flavonoid, saponin, and polysaccharide levels in Sanqi flowers (Li et al., 2025b), and their VOC profiles (mainly terpenes, alkynes, and aromatic hydrocarbons) contribute to the distinctive fragrance (Chen X. F. et al., 2022). Terpenoid compounds, due to their diverse fragrances and biological activities, have extensive applications in the food industry, such as being used as natural flavourings and essences, antioxidants and preservatives, and functional food ingredients (Fan et al., 2023; Carson et al., 2006). Furthermore, Terpenes in Sanqi flowers from the forest understorey also have medicinal value: 3-carene, Germacrene D, and spatulenol modulate antibacterial activity, defense mechanisms, pollination, and anti-inflammation, respectively (Li et al., 2021), while α-guaiene and β-copaene relieve cough and phlegm (Lv et al., 2005) and exhibit antioxidant properties (Usman and Ismaeel, 2020). Different varieties and cultivation practices significantly influence the biosynthesis of VOCs and flavonoids in Sanqi flowers (Chen X. F. et al., 2022; Li et al., 2024b). However, despite the recognized importance of VOCs in Sanqi flowers, there is a significant lack of comprehensive research into how flowering periods and cultivation systems affect VOCs.

Flowering stages and geographical environments significantly shape floral fragrance profiles, with VOC variation across blooming stages driven by physiological, genetic, and environmental factors (Ru et al., 2025; Wang et al., 2025; Song et al., 2025; Du et al., 2022). On one side, enzyme activity and metabolic adjustments mediate VOC synthesis and release; on the other, gene regulatory networks control flowering timing to ensure favorable blooming conditions (Li et al., 2024c). For instance, transcription factors such as those from the MADS-box gene family play crucial roles in floral organ development (Li et al., 2023). Simultaneously, Aromatic compounds vary across flowering stages and flower sections, reflecting the plant’s adaptive pollinator attraction strategies (Zhou et al., 2024). Furthermore, abiotic factors such as climate (including temperature, humidity, and light), soil composition, altitude, and nutrient availability significantly influence the synthesis and release of VOCs (Chen X. F. et al., 2022; Li et al., 2024a), as VOCs are temperature-sensitive and drive dynamic changes in floral components. The emission rate of VOCs accelerates with rising temperatures (Hu et al., 2023; Fu et al., 2017), but this relationship is not a simple linear increase (Sagae et al., 2008). Previous studies have revealed that the VOCs of Sanqi flowers are significantly influenced by genetic factors (Chen X. F. et al., 2022). However, limited information was available regarding the mechanisms by which abiotic factors affect the synthesis and release of VOCs. Therefore, in-depth research into the impact of abiotic factors on the VOCs of Sanqi flowers holds significant theoretical value and practical application significance for optimizing cultivation environments and enhancing medicinal quality.

Organic rather than conventional management of Sanqi not only improves its quality (Li et al., 2024b), but also alter soil metabolites (He et al., 2025), increase microbial diversity (Zhao et al., 2025; Hei et al., 2025), enhance soil fertility, and reduce heavy metal content (Liu et al., 2025). Moreover, *Pinus armandii* and *Pinus yunnanensis* are primarily suitable for Sanqi cultivation. However, the specific impacts of Sanqi-*P. armandii* (SPA) and Sanqi-*P. yunnanensis* (SPY) agroforestry systems on the VOCs in Sanqi flowers are not yet fully understood. Thus, these two systems were the focus of this study. By integrating headspace-solid-phase micro-extraction (HS-SPME) technology with gas chromatography-mass spectrometry (GC), the characteristics of VOCs in Sanqi flowers were systematically analyzed across flowering periods and cultivation practices. The objective of this study is twofold: (1) to investigate the effects of flowering periods (unopened, half-opened, fully opened stages) and two distinct agroforestry systems (SPY and SPA) on the composition, content, and characteristic VOCs of Sanqi flowers, and to unravel the key environmental factors and their interaction mechanisms regulating VOC biosynthesis and emission; (2) to provide practical guidance for optimizing cultivation practices and determining the optimal harvesting time of Sanqi flowers, thereby supporting the efficient utilization of Sanqi flowers in functional foods, beverages, and natural food additives with enhanced sensory and medicinal-edible qualities.

## 2 Materials and methods

### 2.1 Study site and sample collection

The SPA and SPY bases are located in Xundian Hui and Yi Autonomous County, Kunming City (103°11′17″E, 25°27′15″N) and Boshang Town, Linxiang District, Lincang City (100°7′12″E, 23°45′40″N), respectively. Information on the cultivation of Sanqi in forest understories can be found in previous studies (Hei et al., 2025; He et al., 2025). Initially, Sanqi seeds were sown in a greenhouse to ensure healthy growth. In December 2019, after the Sanqi plants had matured for one year, ridge operations were conducted in the *P. armandii* and *P. yunnanensis* forests. The ridges were constructed with a height of 30–40 cm, a width of 80–100 cm, and a length of 120–150 cm. Subsequently, the plants were transplanted into the forest understory ridges at a spacing of (10–15 cm) × (10–15 cm). The transplanted Sanqi seedlings were immediately covered with soil to a depth of 3–5 cm, followed by a layer of pine needles with a thickness of 3–5 cm. Additionally, the cultivation of Sanqi was conducted using organic management practices, without the application of pesticides or fertilizers. Sanqi flowers typically bloom in summer, spanning from June to August annually. The Sanqi flower passes through three distinct flowering stages, each with characteristic morphological features: (1) Unopened stage: Buds are small (0.5 cm), light green, and tightly clustered. (2) Half-opened stage: Buds gradually unfurl to ~1.0 cm, displaying a deeper green hue, with their edges beginning to expand and open. (3) Fully opened stage: Central and upper buds expand to 1.5 cm, with an intensified deep green colour (**Supplementary Figure S1**).

A total of 18 plots were established, corresponding to 3 flowering periods (unopened, half opened, and fully opened stages), 2 systems (SPA, and SPY), and 3 replicates. On July 21, 2022, we selected healthy, disease-free Sanqi flowers (2nd year) with uniform growth patterns from the SPA and SPY systems. To minimize the impact of environmental factors on volatile organic compound (VOC) emissions, the collection process was conducted under conditions of no rain, no wind, and clear weather. As the weight of Sanqi flowers varies across different flowering stages, 20, 10, and 7 Sanqi flowers were sampled at the unopened, half-opened, and fully opened stages, respectively, to ensure consistent weight. Subsequently, one flower was collected from each plant within each plot. Each repetition included 20, 10, and 7 Sanqi flowers, respectively. Thus, a total of 111 flowers were collected. Simultaneously, a handheld weather meter was utilized to measure the temperature, humidity, and altitude during each Sanqi flower collection. Upon collection, the flowers were immediately placed into 20-ml headspace glass bottles (transparent, flat-bottomed), each containing approximately 0.4 grams. The samples were then sealed with aluminum stoppers using sealing pliers. Upon arrival at the laboratory, the samples were transferred to a temperature-controlled room set at 25 °C for 30 minutes to allow the VOCs to reach equilibrium before analysis. The VOCs of the Sanqi flowers were measured immediately upon their return to the laboratory.

### 2.2 HS-SPME and GC-MS analysis

HS-SPME analysis was conducted using 100 μm PDMS solid phase microextraction fibers in conjunction with a headspace solid phase microextraction device from Thermo Fisher Scientific (TriPlus 300, Waltham, MA, USA), following previously established determination methods (Chen X. F. et al., 2022). After allowing the Sanqi flowers to equilibrate at room temperature for 30 minutes, their VOCs were extracted to adsorb. Prior to sampling the VOCs, the SPME fiber was conditioned at the GC-MS injection port for 40 minutes at 250 °C. The fiber was then inserted into the top of a sealed SPME vial using an autosampler.

Upon completion of the adsorption phase, the extraction head should be detached and subsequently inserted into the injection port of the GC-MS (Trace GC Ultra/ITQ 900, Thermo Fisher Scientific). After heating to 250 °C for 20 minutes, the instrument began to collect data. The GC operating conditions were as follows: the HP-5MS capillary column had a diameter of 0.25mm, a length of 30m, and a film thickness of 0.25μm; high-purity helium gas (with a purity of 99.999%) was used as the carrier gas, with the flow rate set to 1.0 mL/min. The heating procedure was as follows: Set the injection port temperature to 250 °C. The column was initially heated to 50 °C for 4 minutes, then gradually increased at a rate of 10 °C/min until it reached 150 °C for 15 minutes. Subsequently, the temperature continued to rise to 250 °C, which was maintained for 11 minutes at a rate of 2 °C/min. The MS conditions were as follows: EI ion source at 69.9 eV, ion source temperature at 230 °C, emission current at 34.6 μA; electron multiplier voltage at 1624 V; mass scan range from 10 to 701 amu.

### 2.3 Data and statistical analysis

The National Institute of Standards and Technology (NIST) mass spectrometry database was utilized for the purpose of qualitative identification, and only those results exhibiting positive and negative matching values exceeding 700 were chosen for subsequent analysis. By employing the total ionic current chromatogram, we utilized peak area normalization to ascertain the relative concentrations of the compounds in the sample. Compound identification was primarily based on retention indices (RI), supplemented by confirmation through comparison with published literature and an online library (https://webbook.nist.gov/chemistry/cas-ser.html). Under identical chromatographic conditions, we calculated retention indices (RI*) using n-alkanes (C5-C19) injected into the same instrument, which served as a reference for substance identification (Li et al., 2017). In addition, the histograms were constructed using GraphPad Prism (version 8), while the principal component analysis (PCA) was performed with Origin (version 2021). Heatmap clustering, Redundancy analysis (RDA), and permutation multivariate analysis of variance (PERMANOVA) were all plotted using R software (version 2.5.6).

## 3 Results

### 3.1 Analysis of the types and contents of the VOCs in Sanqi flowers

A total of 60 and 55 VOC types were identified in the SPY and SPA systems, respectively. The VOCs across flowering stages ranked as follows: fully opened stage (19 and 21 types) > half opened stage (18 and 20 types) > unopened stage (18 and 19 types) in the SPA and SPY system, respectively (**Supplementary Table S1**). Notably, three types of VOCs were detected in the Sanqi flowers: sesquiterpenes (30 species), monoterpens (4 species), and others (4 species) (**Supplementary Table S1**). Moreover, sesquiterpenes were more abundant in the SPY system, accounting for 6.49%–22.77%, while monoterpenes were predominant in the SPA system, ranging from 76% to 98.3%. As the predominant compounds in the SPY and SPA systems, Germacrene D and β-ocimene were present at higher concentrations in the SPY system.

**Figure 1:**
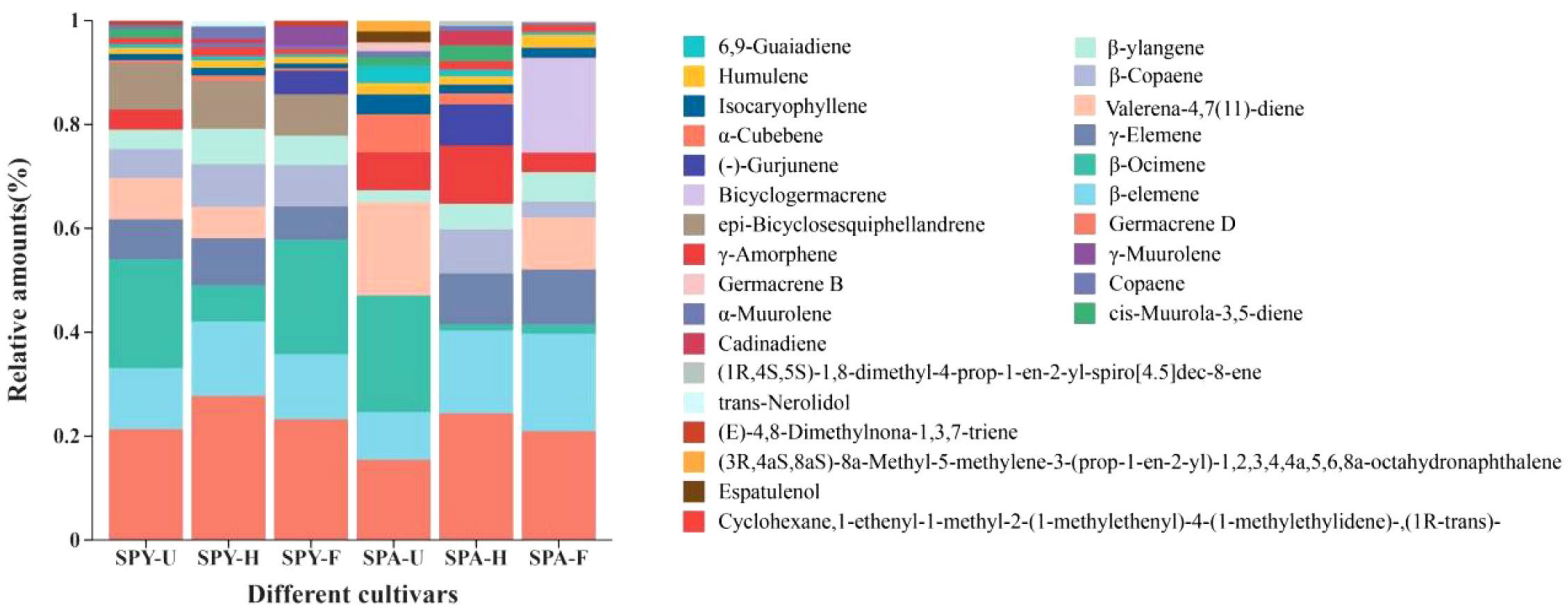
The VOCs in the flowers of Sanqi within the SPY and SPA systems (>1%).

The 27 types of VOCs accounting for over 1% of the total mainly included germacrene D (21.35–26.24% and 15.46–24.41%), β-ocimene (21.28–6.60% and 1.21–22.48%), and β-elemene (11.72–13.52% and 9.15–18.77%), respectively (**Figure 1**). Additionally, β-Ocimene (20.95-22.48%) was abundant in the unopened Sanqi flowers, whereas Germacrene D, accounting for 24.41-26.24% and 20.94-22.51%, was predominant in the half-opened and fully-opened Sanqi flowers, respectively. Therefore, Germacrene D, β-Ocimene, and β-Elemene were the primary compounds found in the Sanqi flowers from the SPA and SPY systems.

### 3.2 Hierarchical clustering analysis of the VOCs in Sanqi flowers

**Figure 2:**
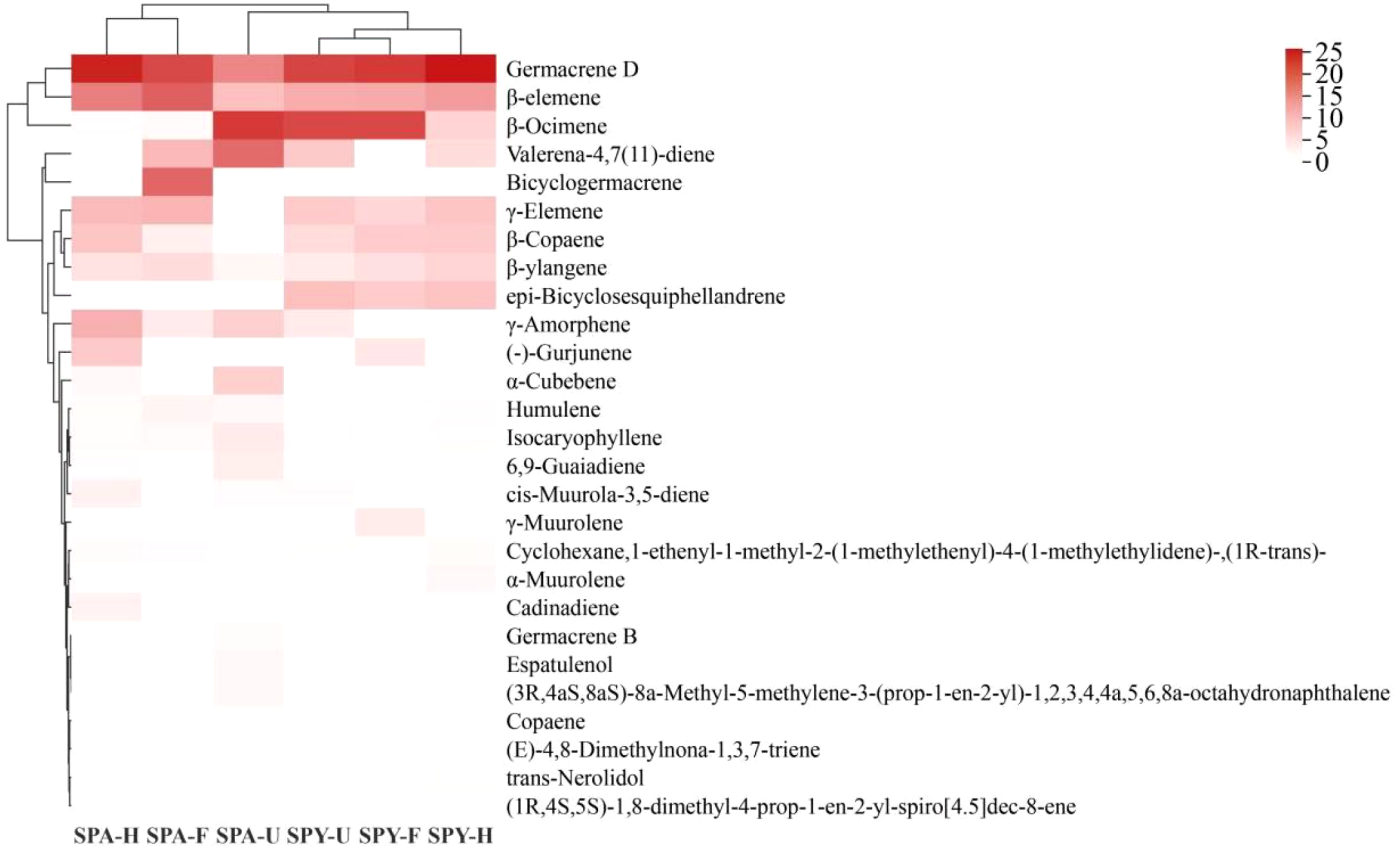
Heat map and hierarchical hierarchical cluster analysis of VOCs in the flowers of Sanqi within the SPY and SPA systems (>1%).

Hierarchical cluster analysis revealed that the VOCs in Sanqi flowers could be categorised into three distinct groups. Within the SPY system, the VOCs formed a single group due to their high content of Germacrene D, β-Ocimene, and β-Elemene. The VOCs from half-opened and fully-opened flowers were predominantly grouped within the SPA system, while the VOCs from unopened Sanqi flowers formed a separate group (**Figure 2**).

### 3.3 Principal component analysis of the VOCs in Sanqi flowers

**Figure 3:**
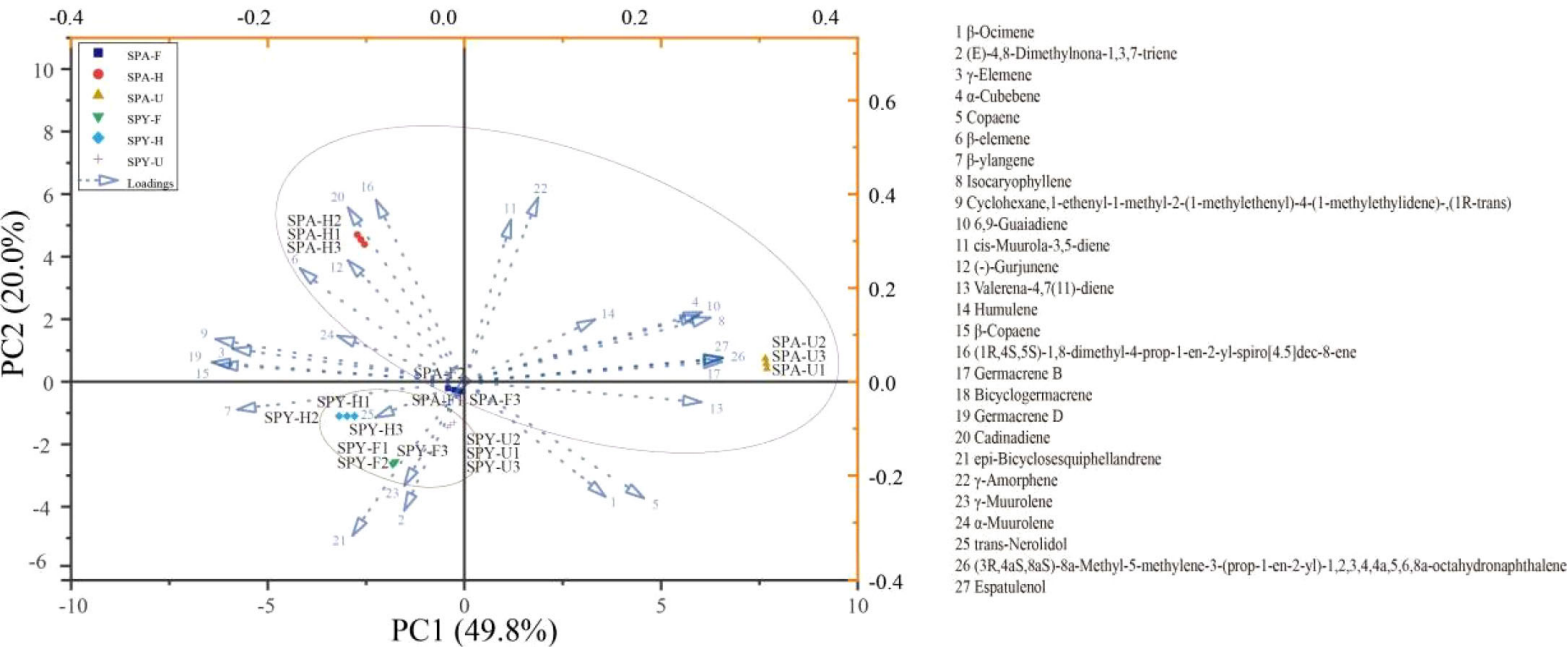
PCA analysis of the VOCs in Sanqi flowers across various flowering periods and cultivation systems.

Principal Component Analysis (PCA) indicated that the variances of PC1 and PC2 accounted for 49.80% and 20.00%, respectively (**Figure 3**). The VOCs in Sanqi flowers were mainly driven by the cultivation system. Moreover, the unique VOCs that differentiate the flowering stages were trans-Nerolidol, (E)-4,8-Dimethylnona-1,3,7-triene, and γ-Muurolene from the PYS system, and Cubenene, Espatulenol, and Bicyclogermacrene from the PAS system. Additionally, the distinctive VOCs in cultivation systems were trans-nerolidol, (E)-4,8-dimethylnona-1,3,7-triene, and γ-muurolene from the PYS system, and (1R,4S,5S)-1,8-dimethyl-4-prop-1-en-2-yl-spiro[4.5]dec-8-ene, germacrene B, and bicyclogermacrene from the PAS system.

### 3.4 Redundancy analysis

**Figure 4:**
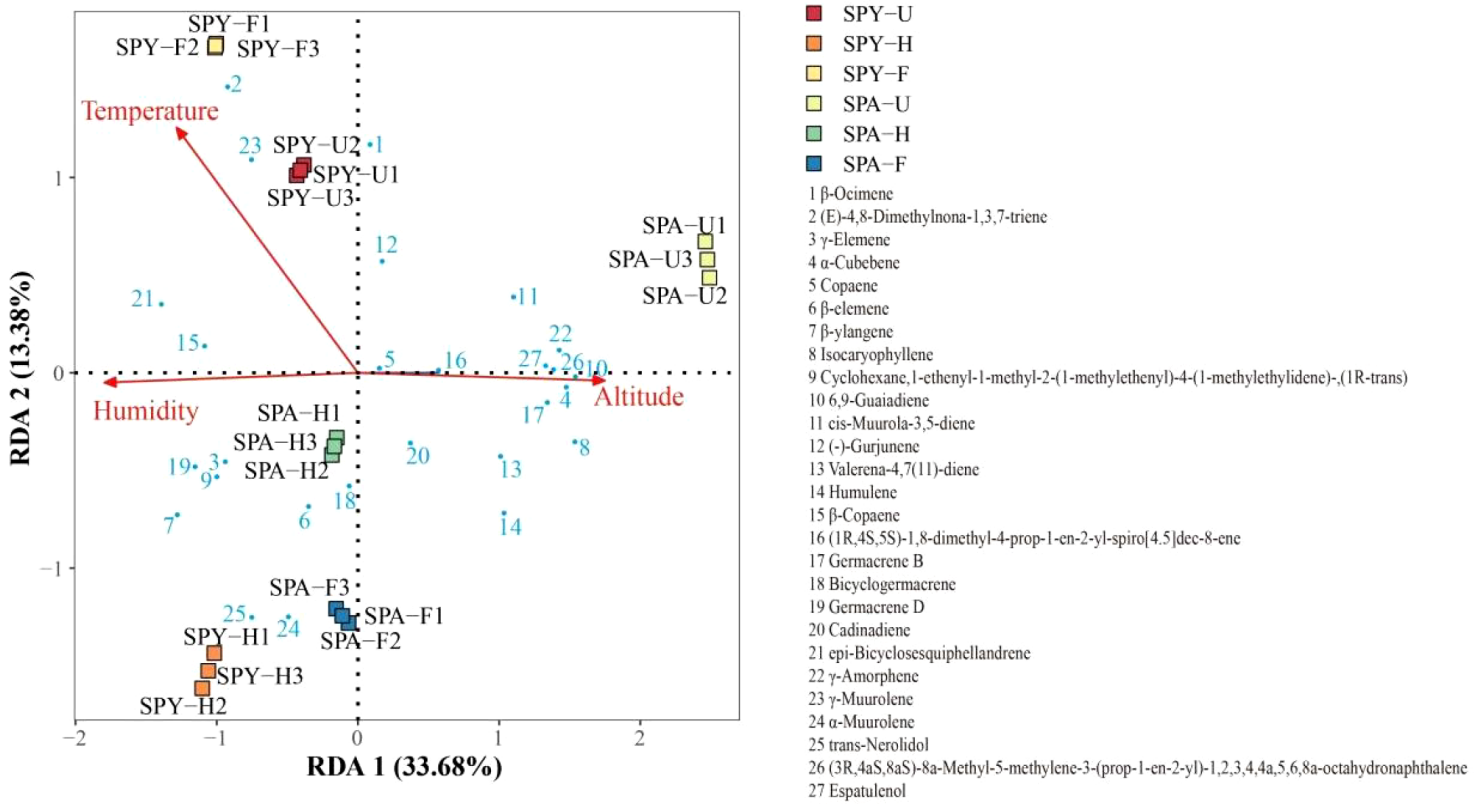
Redundancy analysis (RDA) of the VOCs in Sanqi flowers with environmental factors.

A redundancy analysis (RDA) was conducted to determine the relationship between VOCs and environmental factors (**Supplementary Table S2**). The results indicated that the primary factors influencing the VOCs in Sanqi flowers were humidity, followed by temperature, and altitude (**Figure 4**). Furthermore, humidity and temperature were positively and negatively correlated with (E)-4,8-Dimethylnona-1,3,7-triene, respectively. Conversely, (E)-4,8-Dimethylnona-1,3,7-triene, α-Cubebene, Isocaryophyllene, 6,9-Guaiadiene and γ-Amorphene were negatively and positively correlated with the humidity and temperature (**Supplementary Figure S2**).

## 4 Discussion

A total of 53 VOCs, predominantly terpenoids, have been observed in the flowers of various Sanqi genotypes [3]. Similarly, our research has identified 60 and 55 VOCs in the Sanqi flowers of SPY and SPA agroforestry systems, respectively, with terpenes being the predominant components. Sanqi flowers, when managed with conventional practices, primarily contain various sesquiterpene compounds, such as spathulenol, germacrene D, bicyclic germacrene, and α-panaxine (Li et al., 2017). However, our study revealed elevated levels of germacrene D, β-ocimene, and β-elemene in the Sanqi flowers. This finding contradicts our earlier results, which indicated that 3-carene and germacrene D were the primary VOCs during full flowering (Chen X. F. et al., 2022). On the one hand, organic management practices, which involve no application of pesticides and fertilizers, can improve the soil environment and enhance the volatile VOCs of medicinal herbs (Maggio et al., 2013). On the other hand, Sanqi flowers are typically harvested in the first and second years. Consequently, the timing of these harvests is the primary reason for the variation in germacrene D, β-ocimene, and β-elemene content. β-Ocimene, Germacrene D, and β-Elemene play vital roles in the food industry. These compounds not only enhance food flavor and sensory appeal but also demonstrate antioxidant and antimicrobial properties. For example, β-Ocimene and Germacrene D are widely used in flavor formulation for food and beverages (Kou et al., 2024; Pratama et al., 2022), as well as in spice blends for alcoholic beverages and premium processed foods (Thang et al., 2014; Kazemeini et al., 2021). Meanwhile, β-Elemene serves both as a direct flavor additive in food products and as an essential component in spice mixtures (Chen S. Y. et al., 2022; Sieniawska et al., 2018). Additionally, both β-Ocimene and β-Elemene exhibit remarkable antioxidant properties that effectively inhibit lipid oxidation and free radical formation, which are vital for extending food shelf life and maintaining freshness (Bensid et al., 2020; Xiang et al., 2024). Germacrene D’s broad-spectrum antibacterial properties further enhance food hygiene standards by protecting moist foods from various spoilage-causing bacteria (Thang et al., 2014). Additionally, the compounds β-ocimene, germacrene D, and β-elemene possess medicinal properties. For instance, β-ocimene can induce plant defense responses (Kang et al., 2018), facilitate inter-plant communication (Cascone et al., 2015), enhance crop resistance (Xie et al., 2025), and regulate secondary metabolites (Xiao et al., 2020). Germacrene D plays a vital role in pest and disease resistance (Caselli et al., 2021), interplant communication (Prosser et al., 2004), the composition of essential oils, and antibacterial properties (Rguez et al., 2023). β-Elemene inhibits tumor cell growth and exhibits anti-inflammatory properties. Consequently, functional beverages or dietary supplements containing β-Elemene have been proposed to aid in boosting immunity and may possess further anti-cancer potential (Chen S. Y. et al., 2022). As the demand for natural food additives continues to rise, terpenoid compounds such as β-ocimene, germacrene D, and β-elemene have emerged as promising candidates for next-generation additives (Chen S. Y. et al., 2022). This is thanks to their sustainable potential and natural, plant-derived properties.

The flowering stage significantly affects the types and concentrations of VOCs, with these changes reflecting the plant’s physiological state, ecological functions, and interactions with the environment (Liu J. C. et al., 2023). Our research demonstrated that the VOCs in Sanqi flowers progressively increased during the flowering period, peaking at full bloom. This finding aligns with observations that *Michelia crassipes* petals contain the most volatile components during full opened flowering stage. This is primarily attributed to the emission of VOCs from flowers, which is influenced by the plant’s growth stage, characteristics of floral organs, variations in gene expression, epigenetic modifications, and external environmental conditions (Picazo-Aragonés et al., 2020; Li et al., 2025b). Simultaneously, various flowering stages impact not only the total quantity and composition of VOCs but also yield compounds that are unique to a specific stage. For instance, phenylpropanoids/styrene was abundant in D. chrysotoxum during the initial blooming stage, while isoprenoid-like compounds were more prevalent at the full blooming stage. The concentration of germacrene D in sanqi flowers first increased and then decreased, peaking at the half-bloom stage. Moreover, the highest concentration of β-ocimene was found in unopened Sanqi flowers. This is because different flowering periods regulate the genes responsible for synthesising germacrene D and β-ocimene (Li et al., 2022). In addition, the unique VOCs that differentiate the flowering stages were trans-Nerolidol, (E)-4,8-Dimethylnona-1,3,7-triene, and γ-Muurolene from the PYS system, and Cubenene, Espatulenol, and Bicyclogermacrene from the PAS system. In summary, by utilizing our knowledge of the VOCs emitted at different stages of flowering, we can harvest Sanqi flowers at the optimal time to select specific VOCs for incorporation into food products.

Plants in various geographical regions, even those of the same species, may display significant variations in their floral fragrance components. This phenomenon is attributed to abiotic factors such as climate conditions, soil composition, altitude, and nutrient availability (Campbell et al., 2018; Luizzi et al., 2021; Wu et al., 2024). The PCA analysis revealed that the PYS and PAS systems significantly influenced the content and composition of VOCs, consistent with findings from other studies (Li et al., 2022). High temperatures can enhance the activity of enzymes involved in VOCs synthesis, further accelerating the evaporation of VOCs (Barman and Mitra, 2021). Moreover, humidity significantly influences the quality traits of blackcurrant fruits, including the accumulation of VOCs (Pott et al., 2023). The RDA analysis results further confirmed that humidity being the primary factors affecting the VOCs in Sanqi flowers. Moreover, the humidity were higher in the PYS system than in the PAS system (**Supplementary Table S2**). Hence, higher humidity are key factors influencing the VOCs in the flowers of Sanqi, since the PYS system maintains higher longer rainy seasons. Meanwhile, the composition and activity of soil microbial communities significantly influence plant growth and metabolism, which in turn affects the production of VOCs (Sun et al., 2020). Research has shown that the composition of soil microbial communities is influenced by the species of pine employed in the Sanqi-pine agroforestry system. Furthermore, endophytic bacteria can be transmitted from pine trees to Sanqi plants, thereby influencing the release of VOCs (Jia et al., 2022; Li et al., 2024b). Additionally, the availability of nutrients in the soil influences the phenotypic plasticity of VOCs in the plant (Li et al., 2025a). In conclusion, the composition and content variations of VOCs in Sanqi flowers result from the combined effects of abiotic factors (such as humidity and temperature) and biotic factors (like soil microorganisms and endophytes). These factors regulate Sanqi metabolic pathways and enzyme activities, ultimately leading to the distinct characteristics of VOCs in different geographical regions or agroforestry systems. Future research could further integrate transcriptomic and metabolomic approaches to elucidate the molecular mechanisms underlying how humidity, temperature, and microorganisms influence the VOCs synthesis in Sanqi flowers. This would provide theoretical foundations for optimizing product quality through environmental regulation or microbial intervention strategies.

## 5 Conclusions

A total of 60 and 55 VOCs were identified in Sanqi flowers from the SPY and SPA agroforestry systems, respectively, with terpenoids as the predominant component class. Germacrene D and β-ocimene were more abundant in the SPY system, whereas β-elemene was richer in the SPA system. The cultivation system was the primary factor shaping VOC composition, while humidity (followed by temperature) was the key environmental driver. Understanding the variations in VOC profiles across flowering periods and cultivation regimes offers practical guidance for optimizing Sanqi flower harvesting, supporting their effective utilization in functional foods.

## Data Availability

The original contributions presented in the study are included in the article/**Supplementary Material**. Further inquiries can be directed to the corresponding authors.
