# Supplementary material for: Effects of flowering period and cultivation practices on volatile organic compounds in Sanqi flowers
Source: Front Plant Sci. 2026 Feb 6;17:1761183. doi: 10.3389/fpls.2026.1761183 (PMC12920576; doi:10.3389/fpls.2026.1761183)
Supplement: Supplementary file 1 [file DataSheet1.docx]

***Supplied Materials***

**Effects of Flowering Period and Cultivation Practices on Volatile Organic Compounds in Sanqi Flowers**

**Fan Yang^1†^, Yue Li^1†^, Tongning Yi^2†^, Jingying Hei^1^, Biao Wang^3*^, Xiahong He^4*^, Shu Wang^1*^**

^1^ College of Landscape Architecture and Horticulture, Southwest Forestry University, Kunming 650224, China.

^2^ Department of Endocrinology, the Affiliated Hospital of Liaoning University of Traditional Chinese Medicine, Shenyang 110031, China.

^3^ Department of Biochemistry and Molecular Biology, School of Life Sciences, China Medical University, Shenyang 110122, China.

^4^ Yunnan Provincial Key Laboratory for Conservation and Utilization of In-forest Resource, Southwest Forestry University, Kunming, 650224, China.

^†^These authors contributed equally to this work.

***Correspondence:**

wangbiao@cmu.edu.cn (B. W); wangshu@swfu.edu.cn (S.W.); hxh@swfu.edu.cn (X.H.)

**Table S1.** Relative content of VOCs in of Sanqi flowers under various flowering periods and planting systems

| **CAS** | **Time** | **RI** | **RI*** | **Compounds** | **Relative Contents (%) ± SD** | | | | | |
| --- | --- | --- | --- | --- | --- | --- | --- | --- | --- | --- |
|  |  |  |  |  | **SPY-U** | **SPY-H** | **SPY-F** | **SPA-U** | **SPA-H** | **SPA-F** |
| 3779-61-1 | 10.18 | 1039 | 1038 | trans-β-Ocimene | − | − | 0.41±0.16 | − | − | − |
| 13877-91-3 | 10.39 | 1048 | 1049 | β-Ocimene | 20.78±0.09 | 6.49±0.11 | 20.85±0.42 | 22±0.93 | 1.2±0.05 | 1.76±0.13 |
| 19945-61-0 | 11.58 | 1117 | 1115 | (E)-4,8-Dimethylnona-1,3,7-triene | 0.79±0.04 | − | 1.00±0.02 | − | − | − |
| 7216-56-0 | 11.84 | 1131 | 1130 | Neo-alloocimene | − | − | 0.50±0.11 | − | − | − |
| 3242-08-8 | 15.19 | 1418 | 1415 | γ-Elemene | 7.64±0.28 | 8.44±0.64 | 6.10±0.47 | − | 9.75±0.56 | 10.54±0.57 |
| 17699-14-8 | 15.45 | 1385 | 1421 | α-Cubebene | 0.44±0.13 | 0.87±0.05 | 0.44±0.04 | 7.16±0.91 | 2.08±0.35 | 0.1±0.02 |
| 14912-44-8 | 15.93 | 1406 | 1432 | Ylangene | − | − | − | 0.76±0.13 | − | − |
| 3856-25-5 | 16.05 | 1432 | 1435 | Copaene | 0.82±0.08 | 0.33±0.11 | 0.60±0.08 | 1.14±0.04 | − | 0.38±0.11 |
| 33880-83-0 | 16.13 | 1445 | 1436 | (-)-β-elemene | 0.41±0.10 | 0.81±0.00 | 0.58±0.12 | − | 0.58±0.09 | − |
| 515-13-9 | 16.28 | 1421 | 1440 | β-elemene | 11.63±0.33 | 13.29±0.21 | 11.86±0.48 | 8.95±0.52 | 15.8±0.67 | 18.67±0.16 |
| 110823-68-2 | 16.35 | 1449 | 1441 | 1-ethenyl-1-methyl-2,4-bis(1-methylethenyl)-Cyclohexane | − | − | − | 0.48±0.06 | − | − |
| 62338-43-6 | 16.68 | - | 1449 | 1,2,3,6-tetramethyl-Bicyclo[2.2.2]octa-2,5-diene | − | − | − | − | − | 0.2±0.03 |
| 6831-16-9 | 16.7 | 1428 | 1449 | (-)-Aristolene | 0.43±0.06 | − | − | − | − | − |
| 20479-06-5 | 16.99 | 1433 | 1456 | β-ylangene | 3.70±0.18 | 6.31±0.47 | 5.35±0.19 | 2.23±0.03 | 4.89±0.21 | 5.64±0.5 |
| 118-65-0 | 17.05 | 1461 | 1457 | Isocaryophyllene | 1.25±0.09 | 1.37±0.10 | 0.92±0.05 | 3.78±0.33 | 1.68±0.03 | 1.91±0.18 |
| 29873-99-2 | 17.16 | 1465 | 1460 | Cyclohexane,1-ethenyl-1-methyl-2-(1-methylethenyl)-4-(1-methylethylidene)-,(1R-trans)- | 1.17±0.07 | 1.56±0.21 | 0.96±0.08 | − | 1.62±0.13 | 1.31±0.1 |
| 36577-33-0 | 17.50 | 1442 | 1467 | 6,9-Guaiadiene | 0.78±0.11 | 0.76±0.15 | 0.57±0.08 | 3.23±0.3 | 1.31±0.12 | 0.56±0.09 |
| 0-0-0 [53] | 17.65 | 1472 | 1471 | cis-Muurola-3,5-diene | 1.67±0.21 | − | − | 1.66±0.01 | 3±0.22 | 0.11±0.04 |
| 489-40-7 | 17.73 | 1475 | 1472 | (-)-Gurjunene | − | − | 4.27±0.31 | − | 7.91±0.26 | − |
| 351222-66-7 | 17.76 | 1465 | 1473 | Valerena-4,7(11)-diene | 7.86±1.00 | 5.60±0.44 | − | 17.58±0.19 | − | 9.93±0.02 |
| 6753-98-6 | 17.94 | 1477 | 1477 | Humulene | 1.12±1.00 | 1.33±0.09 | 1.12±0.06 | 2.10±0.49 | 1.58±0.13 | 2.46±0.13 |
| 18252-44-3 | 18.13 | 1459 | 1481 | β-Copaene | 5.57±0.13 | 7.69±0.83 | 7.59±0.23 | − | 8.47±0.62 | 3.05±0.12 |
| 24048-44-0 | 18.47 | 1475 | 1489 | (1R,4S,5S)-1,8-dimethyl-4-prop-1-en-2-yl-spiro[4.5]dec-8-ene | − | − | − | − | 1.02±0.13 | − |
| 15423-57-1 | 18.49 | 1535 | 1490 | Germacrene B | − | − | − | 1.67±0.45 | − | 0.38±0.12 |
| 24703-35-3 | 18.5 | 1493 | 1490 | Bicyclogermacrene | − | − | − | − | − | 18.08±0.38 |
| 23986-74-5 | 18.64 | 1496 | 1493 | Germacrene D | 21.18±0.85 | 25.79±0.88 | 22.05±0.73 | 15.13±0.93 | 24.28±0.47 | 20.83±1.06 |
| 267665-20-3 | 18.72 | 1458 | 1495 | Cadina-3,5-diene | − | − | − | − | − | 0.40±0.06 |
| 29837-12-5 | 18.88 | 1503 | 1498 | Cubenene | − | 0.81±0.26 | − | − | 2.76±0.57 | − |
| 62376-14-1 | 18.94 | 1481.00 | 1480.82 | bicyclo[2.2.2]oct-2-ene,1,2,3,6-Tetramethyl- | − | − | − | 0.96±0.08 | − | − |
| 88-84-6 | 18.96 | 1498 | 1500 | β-Guaiene | − | 0.50±0.10 | − | − | − | − |
| 54324-03-7 | 18.98 | 1499 | 1500 | epi-Bicyclosesquiphellandrene | 8.97±0.13 | 8.64±0.92 | 7.57±0.35 | − | − | − |
| 6980-46-7 | 19.26 | 1496 | 1508 | γ-Amorphene | 3.83±0.78 | 5.76±0.40 | 3.15±0.33 | 7.13±0.98 | 11.14±0.67 | 3.75±0.19 |
| 30021-74-0 | 19.57 | 1515 | 1516 | γ-Muurolene | − | − | 3.57±0.10 | − | − | − |
| 24406-05-1 | 20.37 | 1533 | 1536 | α-Cadinene | − | − | 0.60±0.01 | − | − | − |
| 10208-80-7 | 20.38 | 1517 | 1536 | α-Muurolene | − | 2.17±0.05 | − | − | 0.98±0.32 | − |
| 40716-66-3 | 21.09 | 1551 | 1554 | trans-Nerolidol | − | 1.06±0.22 | − | − | − | − |
| 212394-95-1 | 22.02 | 1477 | 1578 | (3R,4aS,8aS)-8a-Methyl-5-methylene-3-(prop-1-en-2-yl)-1,2,3,4,4a,5,6,8a-octahydronaphthalene | − | − | − | 2.04±0.16 | − | − |
| 6750-60-3 | 30.19 | 1622 | 1714 | Espatulenol | − | − | − | 2.05±0.23 | − | − |
| Monoterpene  Sesquiterpene  Other  Total | | | | | 21.57±0.13 | 6.49±0.11 | 22.769±0.71 | 22±0.93 | 1.2±0.05 | 1.76±0.13 |
|  |  |  |  |  | 78.47±5.53 | 91.69±5.59 | 77.3±3.71 | 76±5.61 | 96.09±4.98 | 98.3±3.85 |
|  |  |  |  |  | − | 1.87±0.48 | − | 2.05±0.23 | 2.76±0.57 | 0.2±0.03 |
|  |  |  |  |  | 19 | 20 | 21 | 18 | 18 | 19 |

“−”indicates that the VOCs has no peak.

**Table S2**. Changes in environmental factors of the Sanqi flowers.

|  | **SPY-U** | **SPY-H** | **SPY-F** | **SPA-U** | **SPA-H** | **SPA-F** |
| --- | --- | --- | --- | --- | --- | --- |
| Temperature | 17.87±0.15cd | 16.87±0.51d | 16.47±0.06d | 24.00±0.46a | 22.17±1.08b | 18.60±0.7c |
| Humidity | 0.93±0.06ab | 0.98±0.02a | 0.98±0.00ab | 0.72±0.02d | 0.82±0.05c | 0.89±0.02bc |
| Altitude | 2181±14.13b | 2074±17.67c | 2229±1.43a | 2016±2.08d | 2063±0.58c | 2077±2.89c |

Different lower-case letter indicates significant difference at p<0.05.


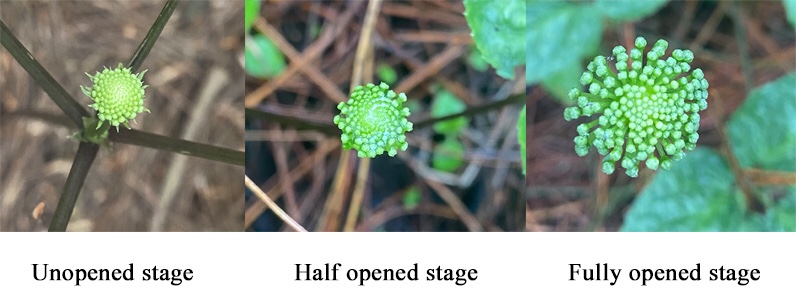


**Figure S1**. Morphological characteristics of Sanqi flowers


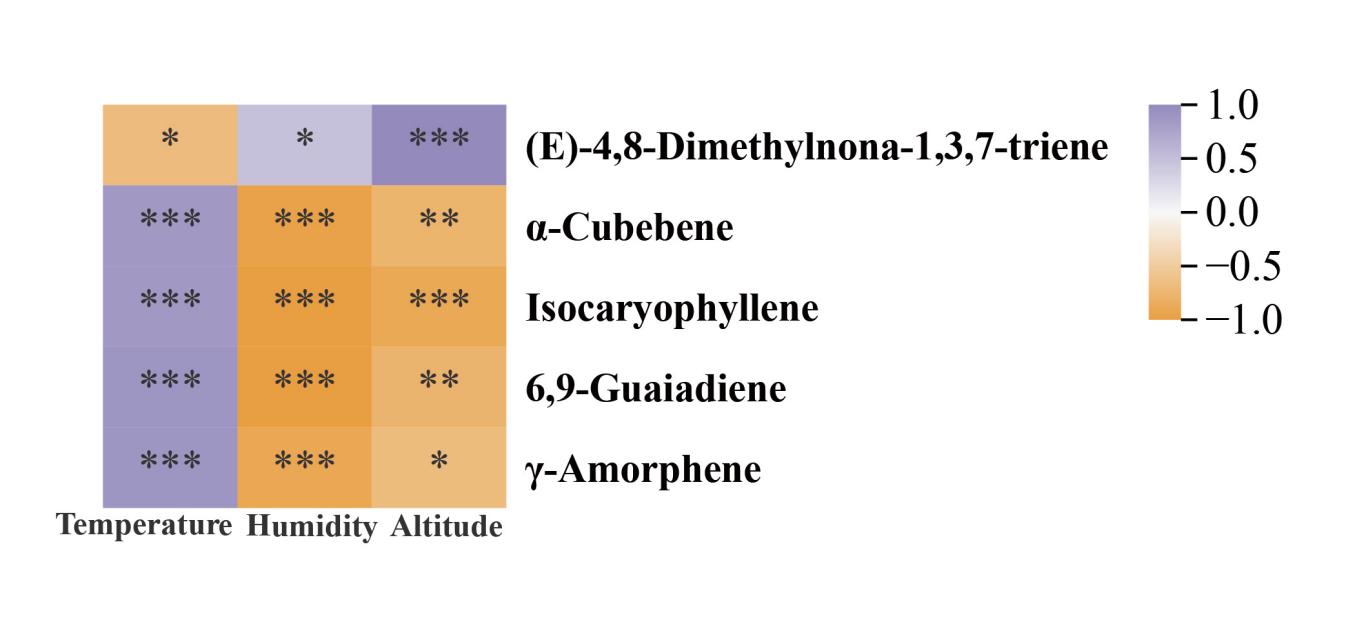


**Figure S2**. Correlation between VOCs and environmental factors in Sanqi flowers.
